# O-GlcNAcylated RALY Contributes to Hepatocellular Carcinoma Cells Proliferation by Regulating USP22 mRNA Nuclear Export

**DOI:** 10.7150/ijbs.97397

**Published:** 2024-07-01

**Authors:** Shiwei Liu, Qingpeng Lv, Xinyu Mao, Hui Dong, Wenjing Xu, Xuanlong Du, Weilu Jia, Kun Feng, Jiaqi Zhang, Yewei Zhang

**Affiliations:** 1 School of Medicine, Southeast University, Nanjing 210009, China.; 2 Hepatopancreatobiliary Center, The Second Affiliated Hospital of Nanjing Medical University, Nanjing 210011, China.

**Keywords:** hepatocellular carcinoma, mRNA nuclear export, O-GlcNAcylation, peptide proteolysis-targeting chimera, RALY

## Abstract

Hepatocellular carcinoma (HCC) is one of the most prevalent and deadly tumors; however, its pathogenic mechanism remains largely elusive. In-depth researches are needed to reveal the expression regulatory mechanisms and functions of the RNA-binding protein RALY in HCC. Here, we identify RALY as a highly expressed oncogenic factor that affects HCC cells proliferation both *in vitro* and *in vivo*. O-GlcNAcylation of RALY at Ser176 enhances its stability by protecting RALY from TRIM27-mediated ubiquitination, thus maintaining hyper-expression of the RALY protein. Mechanistically, RALY interacts with USP22 messenger RNA, as revealed by RNA immunoprecipitation, to increase their cytoplasmic localization and protein expression, thereby promoting the proliferation of HCC cells. Furthermore, we develop a novel RALY protein degrader based on peptide proteolysis-targeting chimeras, named RALY-PROTAC, which we chemically synthesize by linking a RALY-targeting peptide with the E3 ubiquitin ligase recruitment ligand pomalidomide. In conclusion, our findings demonstrate a novel mechanism by which O-GlcNAcylation/RALY/USP22 mRNA axis aggravates HCC cells proliferation. RALY-PROTACs as degraders of the RALY protein exhibit potential as therapeutic drugs for RALY-overexpressing HCC.

## Introduction

Liver cancer remains the main contributor to the global cancer burden. Hepatocellular carcinoma (HCC), the primary type of liver cancer, is responsible for the majority of deaths related to liver cancer 1. Contracting hepatitis B and C viruses significantly increase the risk of hepatocarcinogenesis 2. Despite improvements in HCC diagnosis and intervention, the prognosis remains suboptimal. Therefore, in-depth research on the molecular regulatory mechanisms underlying HCC occurrence and clinical progression is required to develop better treatment options and improve clinical outcomes.

Post-translational modifications (PTMs) refer to the covalent and chemical modifications of protein residues that enable cells to respond precisely and quickly to internal and external stimuli through the direct and dynamic regulation of protein functions 3. O-GlcNAc modification (O-GlcNAcylation), a type of PTMs, refers to the process of adding O-linked β-N-acetylglucosamine (O-GlcNAc) via an O-linkage to the serine (Ser) or threonine (Thr) residues of intracellular proteins 4. O-GlcNAc transferase (OGT) exclusively regulates the addition of O-GlcNAc, while glycoside hydrolase O-GlcNAcase controls the removal 5. Aberrant OGT and O-GlcNAcylation have been observed in various tumors and are associated with tumorigenesis and cancer progression 6. Growing evidence indicates that numerous classes of O-GlcNAcylated proteins play a role in the regulation of core processes, including signal transduction, epithelial-mesenchymal transition, and other molecular regulatory mechanisms 7-9. However, the exact mechanism by which O-GlcNAcylation achieves these biological functions and the specific proteins modified by O-GlcNAc that may act as pivotal contributors remain elusive.

RALY belongs to the heterogeneous nuclear ribonucleoprotein (hnRNP) family, comprising a vast array of RNA-binding proteins (RBPs) involved in various processes, from the early steps of RNA biogenesis to cellular localization and transport 10. RALY is elevated in many cancer cells and participates in tumor progression through both proliferation and aggressive biological behaviors 11-13. Moreover, RALY is regulated by PTMs such as phosphorylation and SUMOylation 14, 15. However, the expression regulatory mechanisms of RALY are unclear and how RALY controls the progression of HCC is still largely a mystery.

Herein, we firstly investigate the detailed molecular mechanisms of RALY overexpression. We find that OGT interacts with RALY and catalyzes its O-GlcNAcylation, which inhibits RALY ubiquitination and enhances its stability. In downstream mechanisms, we find that RALY, as an RBP, confers specificity for recruiting USP22 messenger RNA (mRNA) to Aly/REF export factor (ALYREF) and efficiently promotes its export, thereby promoting the proliferation of HCC cells. Furthermore, we develop peptide-based PROTACs (p-PROTACs) that degrade RALY by promoting the recruitment of E3 ubiquitin to RALY. Collectively, our study provides previously unknown mechanistic insights into the relationship between O-GlcNAcylation and RALY protein stability, and reveals the crucial role of aberrant RALY in HCC progression.

## Methods

### Cell culturing

Human hepatocarcinoma cell lines (Hep3B and Huh7) and human hepatic epithelial cell line THLE-2 were purchased from Pricella Company (Wuhan, China). MHCC-97H and MHCC-97L were stored in our laboratory. HCC cells were grown in DMEM with 10% FBS, along with 1% penicillin and 1% streptomycin.

Thiamet-G (TMG) and PUGNAc (PUG), inhibitors of O-GlcNAcase, were introduced into DMEM at final concentrations of 20 μmol/L and 100 μmol/L for 12 hours, respectively. The final concentration of NH4CL, 3-Methyladenine (3-MA), Z-VAD-FMK and MG132 were 10 μmol/L.

The lipofectamine 2000 reagent (11668-027, Thermo Fisher Scientific) was used to transiently transfect the cells on the basis of the manufacturer's guidelines. The target sequences of siRNAs were as follows: 5′-AGACCATCTTCTCTAAGTA-3′ (siRALY#1), 5′-GCAGCATCTGCCATATACA-3′ (siRALY#2), 5′-GAGTCTTCATTGGAAACCT-3′ (siRALY#3), 5′-TGAGCAGTATTCCGAGAAA-3′ (siOGT) and 5′-AGCTACCAGGAGTCCACAAAG-3 (siUSP22). Plasmid wild-type RALY and mutants, including RALYKO-T151A, RALYKO-T175A and RALYKO-S176A, were chemically constructed by Generalbiol Company (Chuzhou, China) and subcloned into the pcDNA3.1 expression vector.

### Western blotting

Total protein was isolated by RIPA Lysis Buffer containing PMSF (P0013B and ST505, Beyotime Biotechnology). Equivalent quantities of proteins were placed into 4-20% SDS-PAGE gels and then moved to polyvinylidene fluoride membranes. First antibodies were used to probe the membranes, followed by incubation with secondary antibodies.

The first antibodies used were mouse anti-O-GlcNAcylation (ab2739, Abcam), anti-β-actin and anti-RALY (HRP-60008 and 68011-1-Ig, Proteintech Group), rabbit anti-USP22, anti-ubiquitin, anti-HNRNPC, anti-CHD4, anti-HNRNPA2B1, anti-PCBP2, anti-STIP1, anti-OGT and anti-TRIM27 (55110-1-AP, 10201-2-AP, 11760-1-AP, 14173-1-AP, 14813-1-AP, 15070-1-AP, 15218-1-AP, 11576-2-AP and 12205-1-AP, Proteintech Group). Second antibodies (SA00001-1 and SA00001-2 from Proteintech Group and A25022 from Abbkine) were acquired for the experiment.

### Cell proliferation assays

For Cell Counting Kit 8 (CCK-8), approximately 1500 cells/well were seeded in a 96-well plate. At the specific time point, CCK-8 solution from Beyotime Biotechnology (C0037) was introduced into the wells and left to incubate for 2 hours. The spectrometer was then used to measure the optical density at a wavelength of 450 nm (OD450).

EdU incorporation assays were carried out using 24-well plates following the guidelines provided by the manufacturer of an EdU kit (C0071S, Beyotime Biotechnology) with Alexa Fluor 488 and Hoechst 33342 reagent (nuclear counterstain). The results were captured under a fluorescence microscope and shown as the ratio of the EdU-positive cells to Hoechst-positive cells.

### Co-immunoprecipitation (CO-IP)

CO-IP was carried out according to the protocol of the rProtein A/G Magnetic IP/Co-IP Kit (AM001, Nanjing ACE Biotechnology). The rProtein A/G magPoly beads were mixed with antibodies and incubated at room temperature for 30 minutes, followed by incubation with cell lysates at 4°C overnight. The beads were rinsed thrice using a washing solution. The enriched complex was eluted from the beads with the elution buffer and then subjected to western blotting or mass spectrometry (MS) analysis. The antibodies used for CO-IP were mouse anti-RALY (68011-1-Ig, Proteintech Group) and anti-lgG, rabbit anti-OGT, anti-TRIM27 (11576-2-AP and 12205-1-AP, Proteintech Group) and anti-lgG. Signal-Seeker^TM^ ubiquitination detection kit (BK161-S, Cytoskeleton) provided ubiquitination affinity beads.

### Immunostaining

Cell lines were inoculated in 6-well dishes containing coverslips, fixed with 4% paraformaldehyde (BL539A, Biosharp) for 10 minutes after adhering to the coverslips. Cells were then permeabilized in enhanced immunostaining permeabilization buffer (P0096, Beyotime Biotechnology), washed by PBS solution. The cells were exposed to first antibodies overnight at 4°C, followed by 1.5-hour incubation with second antibodies conjugated with fluorescein at room temperature in the absence of light. The sample were mounted by Antifade Mounting Medium containing DAPI (P0131, Beyotime Biotechnology) and examined with a confocal microscope in dark room.

### Animal studies

To establish a tumor xenograft model, 2 × 10^6^ Huh7 cells bearing lentivirus were injected into the subcutaneous tissue of each 5-week-old male BALB/c mouse. Four weeks after injection, the mice were euthanized and neoplasms were harvested. The neoplasms were weighed, and the volumes were computed as the following formula: volume (mm^3^) = 0.5 × length × width^2^. Lentivirus and negative control transducing Huh7 cells were synthesized by Hanheng Company (Shanghai, China).

To mimic the process of HCC development, male C57BL/6 mice were intraperitoneally injected with 25 mg/kg diethylnitrosamine (DEN) at the age of 14 days to initialize the HCC process. After 2 weeks, carbon tetrachloride (CCl_4_) (0.5 ml/kg body weight, dissolved in corn oil) was intraperitoneally injected twice weekly for 10 weeks. At 16 weeks postpartum, the mice were administered AAV-shRALY or AAV-ctrl via tail vein injection (2 × 10^11^ virus particles/mouse). At 28 weeks of age, mice of two groups were euthanized for further evaluation.

### RNA immunoprecipitation (RIP) and RIP sequencing (RIP-seq)

The Magna RIP^TM^ RNA-Binding Protein Immunoprecipitation Kit (17-701, Millipore) was used for the RIP procedure. In brief, 2 × 10^7^ cells were lysed in complete RIP lysis buffer (0.5 μl protease inhibitor cocktail, 0.25 μl RNase inhibitor and 100μl RIP lysis buffer) for 1 hour at -80°C and centrifuged at 14000rpm for 10 minutes at 4°C. 100 µL supernatant was placed to each beads-antibody complex coated either with antibodies of interest or with negative control antibody in RIP Immunoprecipitation Buffer (35μl EDTA, 5μl RNase inhibitor and 860μl RIP wash buffer) and incubated overnight at 4°C. The beads were then incubated in the proteinase K buffer (15 μl 10 % SDS, 18μl proteinase K and 117μl RIP wash buffer) at 55°C for 30 minutes with shaking to digest the protein. Finally, RNA was isolated using phenol: chloroform: isoamyl alcohol (125:24:1 pH < 5.0) (AP1025, ACMEC) and processed for RIP-seq analysis or qRT-PCR analysis.

The Tiangen Company (Beijing, China) provided assistance in the RIP-seq-related experiments.

### RNA sequencing (RNA-seq) and analysis

Total RNA samples were isolated from sicontrol and siRALY#1 Hep3B cells (three replicates per sample). The transcriptome sequencing libraries were constructed and sequenced by Tiangen Company (Beijing, China). Differential expression analysis of two conditions/groups was carried out by the DESeq2 R package (1.16.1). Genes were considered to be differentially expressed if they had a P-value < 0.05 and |log2(FoldChange)| > 1.

### Isolation and purification of RNA from both the cytoplasm and nucleus

Subcellular fractionation was carried out by Cytoplasmic & Nuclear RNA Purification Kit (NGB-21000, Norgen Biotek). The purified RNA samples were subjected to subsequent qRT-PCR analysis.

### Total RNA extraction and quantitative real-time PCR (qRT-PCR)

RNA extraction was performed with RNApure Tissue & Cell Kit (CW0584, CWBIO) as recommended by the manufacturer. Subsequently, cDNA was generated with HiFiScript cDNA Synthesis Kit (CW2569, CWBIO) in the light of the manufacturer's protocol. Prior to the PCR experiment, template DNA were mixed with UltraSYBR mixture (CW0957, CWBIO), RNase-free Water and the primers. The following primer sequences were used for the indicated genes: RALY-forward, 5-AGACAAGCCCAGTATGCGAGTG-3; RALY-reverse, 5-TTCTGAGTTGGCAAGTGAAATGAGT-3; USP22-forward, 5-CAAGGAGGAGCAGCGAAAAGC-3; USP22-reverse, 5-AAGAAGTCCCGCAGAAGTGGC-3; HNRNPC-forward, 5-GGAAAAGCAGGTGTGAAACGAT-3; HNRNPC-reverse, 5-GGCACTACAGCCCGAGCAATA-3; CHD4-forward, 5-AGCAGTCTTCCTGTATTCCCTTTA-3; CHD4-reverse, 5-ACGGCTGTCCTTGTCACCCA-3; HNRNPA2B1-forward, 5-GCAACCTTCTAACTACGGTCCAAT-3; HNRNPA2B1-reverse, 5-TACCTCTGGGCTCTCATCCTCTC-3; PCBP2-forward, 5-CATAATCGGGCGTCAAGGC-3; PCBP2-reverse, 5-GGGTGGTGGTGAACAGCAGAA-3; STIP1-forward, 5-CGATGATGCCTTACAGTGCTAC-3; STIP1-reverse, 5-TTTCAGTTGAGGGTTATTTGCC-3; U6-forward, 5-CTCGCTTCGGCAGCACA-3; U6-reverse, 5-AACGCTTCACGAATTTGCGT-3; GAPDH-forward, 5-GACACCCACTCCTCCACCTTT-3; GAPDH-reverse, 5-TCTCTTCCTCTTGTGCTCTTGCT-3. The reaction was amplified for 40 cycles with denaturation at 95°C for 15 seconds, annealing or extension at 60°C for 60 seconds.

### Screening of RALY-binging peptides

The phage display technique was used to screen peptides binding to human RALY protein. Briefly, a library of phage-displayed peptides was transferred to a plate coated with RALY, and left for 1 hour. Subsequently, nonbinding phages were washed off by PBST and bound phages were eluted with triethylamine buffer. The eluted phages were further amplified and applied as input for the next biopanning round. Three screening rounds resulted in a notable enrichment of RALY-binding phage clones. The phage clones chosen from the eluted phages of the last selection round were sequenced.

### Peptides and p-PROTACs production

The ChomiX Biotech Company (Nanjing, China) conjugated five peptides obtained from phage library through solid-phase synthesis. FITC and TAT (GRKKRRQRRRPPQQ) cell penetrating peptides were coupled at the N-terminus and C-terminus of the peptides, respectively. In addition, p-PROTAC was also synthesized via Pomalidomide-PEG1-C2-COOH (CAS NO.: 2139348-60-8) linked with the peptide and named RALY-PROTAC.

### Ethics approval and patient consent statement

3 paired HCC and adjacent normal samples were collected from patients who had surgery and obtained written informed consents. The above study with human tissues was approved by the institutional review board of the Second Affiliated Hospital of Nanjing Medical University (approval numbers 2022-KY-192-01).

All mice were maintained in the Center for Laboratory Animals of Southeast University (Nanjing, China). All manipulations involving live mice were carried out according to established ethical guidelines and with the approval of the Animal Care & Welfare Committee at Southeast University (approval numbers 20230703001 and 20230201040).

### Statistical analysis

GraphPad Prism 8.0.1 software was used to conduct statistical analysis and generate graphs. All values tested in triplicate were presented as mean ± standard deviation (SD). Statistical comparisons among groups were estimated either with Student's t-test or analysis of variance. Values were considered significantly different if p < 0.05 (*P < 0.05, **P < 0.01, ***P < 0.001, ****P < 0.0001).

## Results

### Both *in vitro* and *in vivo*, the ablation of RALY exhibits tumor suppressor effects

**Figure 1:**
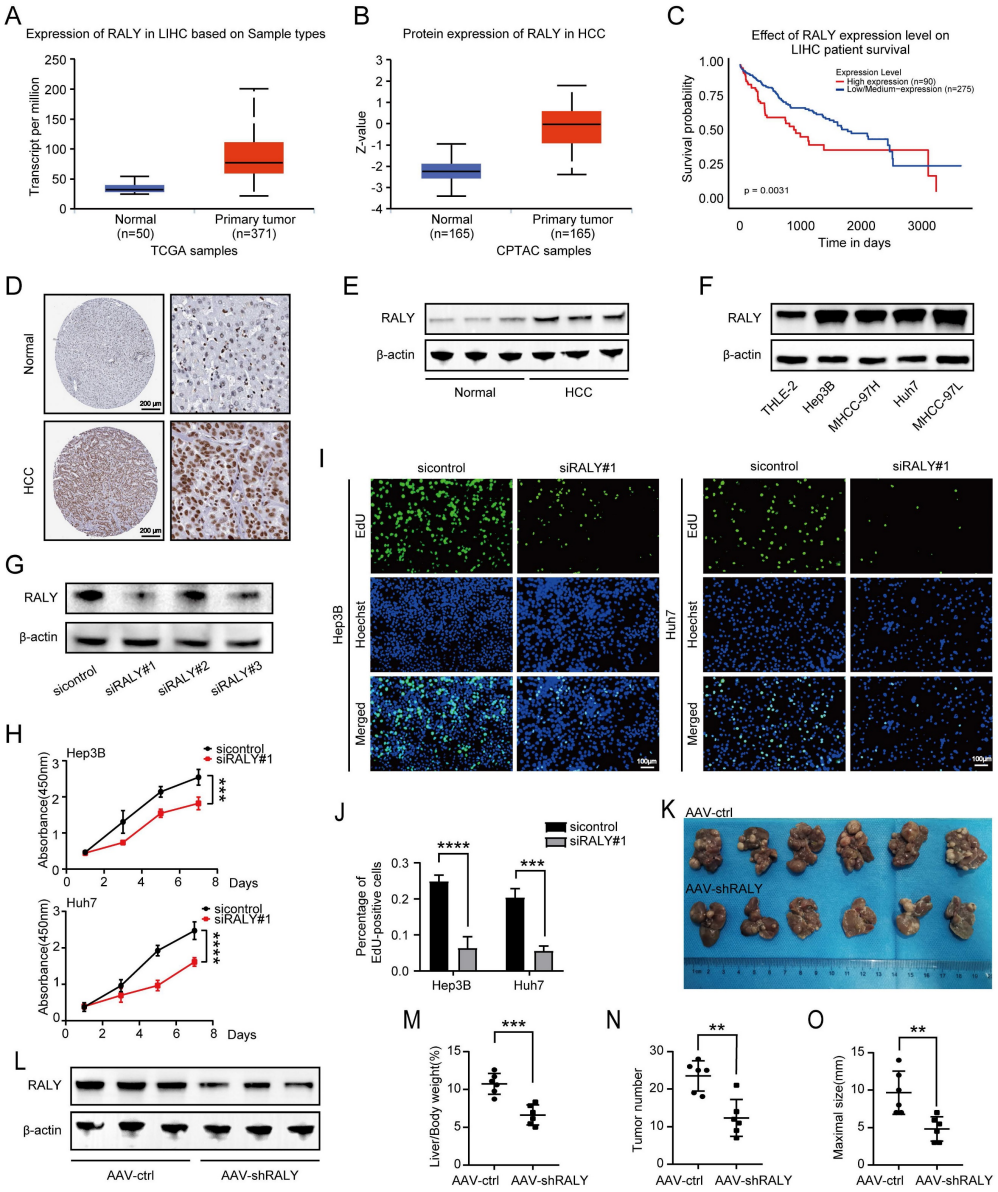
The expression and function of RALY in hepatocellular carcinoma (HCC). (A, B) Bioinformatics analysis predicted RALY mRNA (A) and protein (B) levels in HCC tissues and normal adjacent tissues by UALCAN. (C) Kaplan-Meier analysis was used to measure the survival rate of HCC patients by UALCAN. (D) Immunohistochemistry images of RALY expression in paracancerous tissues and HCC samples were obtained from the Human Protein Atlas database. (E) Western blotting analysis of RALY expression in paracancerous tissues and HCC samples. (F) Western blotting analysis of RALY expression using four HCC cell lines (Hep3B, Huh7, MHCC-97H and MHCC-97L) and normal liver epithelial THLE-2 cells. (G) The expression of RALY protein was detected after RALY knockdown in HCC cells. (H) CCK-8 assays showed the growth rate of HCC cells upon manipulation of RALY. (I) EdU incorporation assays were carried out in HCC cells (Green fluorescence, EdU-positive cells; blue fluorescence, total cells). (J) Statistical analysis of the EdU incorporation assays. (K) Gross appearances of liver samples from the primary liver cancer mouse mode established with C57BL/6 mice. (L) The levels of RALY protein expression in livers from the indicated groups. (M, N, O) Liver-to-body weight ratios (M), tumor nodules numbers (N) and maximal size of tumors (O), n = 6/group.

First, we performed bioinformatics analysis using an online database (UALCAN) to explore the potential role of RALY in HCC 16, 17. The results showed that levels of RALY mRNA and protein were elevated in HCC tissues compared to normal tissues (Figure 1A and 1B). In addition, after evaluating the likelihood of survival for various HCC patients, it was discovered that individuals with elevated levels of RALY had a decreased overall survival compared to those with lower levels of RALY (Figure 1C). Furthermore, our examination revealed that the expression of RALY protein was elevated in HCC specimens when compared to healthy tissues based on the immunohistochemistry data in the Human Protein Atlas database (Figure 1D) and our western blotting outcomes (Figure 1E). Consistent with the conclusion in HCC primary and adjacent normal samples, western blotting revealed increased RALY expression in HCC cell lines (Hep3B, MHCC-97H, Huh7, and MHCC-97L) in comparison to THLE-2 cells (Figure 1F).

To determine the biological effect of RALY on HCC progression, siRNAs (siRALY#1) were used to knockdown RALY; these molecules showed a significant capacity to manipulate RALY expression (Figure 1G). We further evaluated cellular phenotypes following RALY depletion *in vitro*. CCK-8 and EdU assays were performed to determine cell growth and survival (Figure 1H, 1I and 1J), which indicated reduced proliferation capacity in the siRALY group. Next, we used DEN and CCl_4_ to trigger HCC in C57BL/6 mice to validate the *in vivo* results (Figure 1K). The mice were injected with AAV-ctrl or AAV-shRALY via the tail vein, and the manipulation efficiency was confirmed (Figure 1L). Using this model, we determined that RALY ablation led to lower liver-to-body weight ratios, fewer tumor numbers, and smaller tumor sizes (Figure 1M, 1N and 1O). Collectively, these results indicate that RALY is identified as an oncogene in HCC but its abnormal expression and tumor-promoting mechanisms are still unknown.

### RALY is hyper-O-GlcNAcylated at Ser176

**Figure 2:**
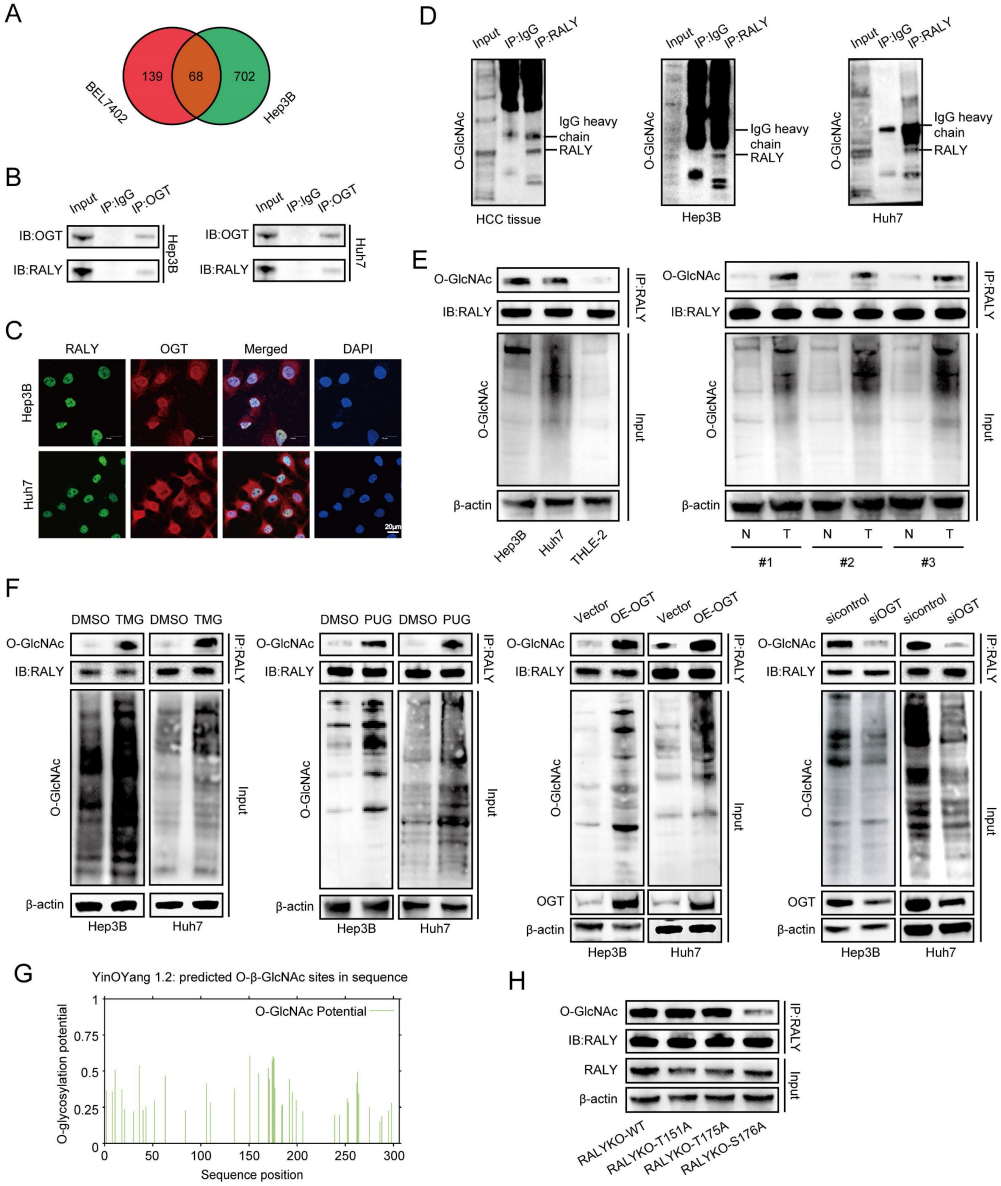
OGT mediated O-GlcNAcylation of RALY on Ser176. (A) Venn diagram illustrating the overlapped proteins bound with OGT in Hep3B and BEL7402 cells. (B, C) Co-immunoprecipitation (CO-IP, B) and immunostaining assays (C) validation of the interaction between RALY and OGT in hepatocellular carcinoma (HCC) cells. (D) CO-IP of O-GlcNAcylated proteins in the immunoprecipitates pulled down by anti-RALY antibodies in HCC tissue and cells. (E) Western blotting showed the expression levels of RALY O-GlcNAcylation using two HCC cell lines (Hep3B and Huh7) and normal liver epithelial THLE-2 cells, and paired HCC specimens and their paracancerous tissues. (F) Western blotting showed the expression levels of RALY O-GlcNAcylation by stimulation and block of O-GlcNAcylation in HCC cells. (G) The predicted O-GlcNAcylation sites of RALY using the YinOYang 1.2 server. (H) Western blotting analyzed the O-GlcNAcylated expression levels of RALY in IP-RALY immunoprecipitates from Hep3B cells transfected with plasmids expressing wild-type or O-GlcNAcylated site mutants.

Many previous studies reported that PTM can regulate RALY, prompting our exploration of more potential PTMs that can modify RALY. In this study, IP-MS analysis indicated binding between RALY and OGT in Hep3B and BEL7402 cells (Figure 2A and Table S1) 18, indicating that RALY may be modified by O-GlcNAcylation, which was further confirmed through CO-IP assays and immunostaining in HCC cells (Figure 2B and 2C). To further clarify whether RALY was O-GlcNAcylated, we conducted CO-IP experiments and observed that O-GlcNAcylated RALY proteins in the immunoprecipitates were captured by anti-RALY antibodies in HCC tissues and cells (Figure 2D). Moreover, RALY showed a stronger O-GlcNAcylation signal in HCC cells compared to normal hepatic cells. Similarly, O-GlcNAcylation of endogenous RALY was strong in HCC tissues (Figure 2E). To further explore the association between O-GlcNAcylation and RALY, we up-regulated global O-GlcNAcylation using stimulators (TMG, PUG and OE-OGT) of O-GlcNAcylation, which resulted in increased RALY O-GlcNAcylation *in vitro*. However, siOGT treatment had the opposite effect (Figure 2F). These findings implied that RALY is highly modified with O-GlcNAc in HCC. To identify the O-GlcNAcylated site(s) of RALY, we predict potential locations through an online database (YinOYang 1.2 Server); Thr151, Thr175, and Ser176 were the most potentially modified sites (Figure 2G). Subsequently, we generated RALY T151A, T175A, and S176A mutants and found that the individual S176A mutants showed reduced RALY O-GlcNAcylation, suggesting that the O-GlcNAcylation site was Ser176 (Figure 2H). Together, these data imply that RALY can bind to OGT and can be hyper-O-GlcNAcylated at Ser176 in HCC.

### O-GlcNAcylation increases the protein stability of RALY

**Figure 3:**
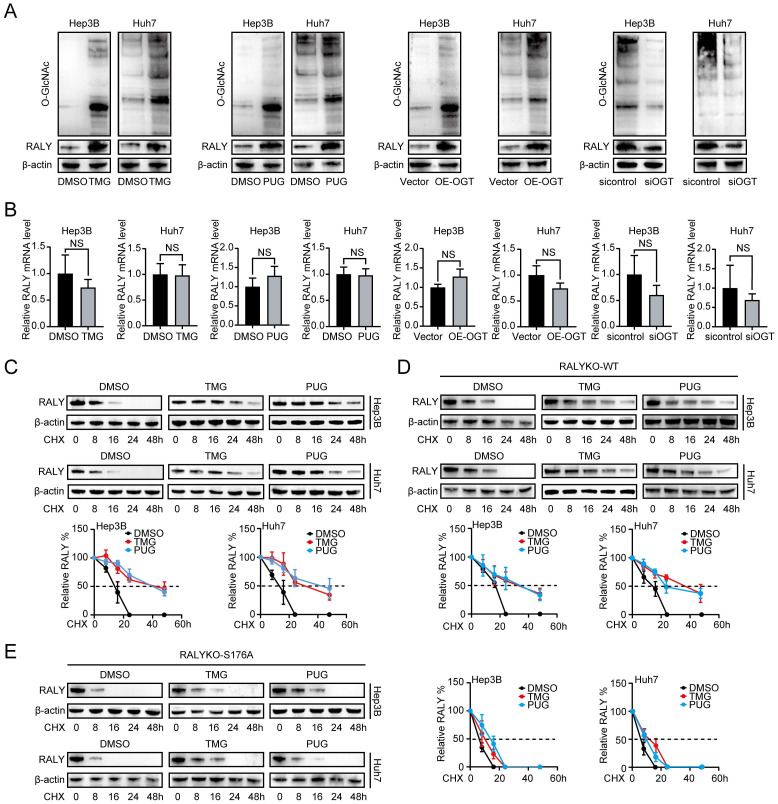
O-GlcNAcylation maintained the protein stability of RALY. (A) RALY was measured by western blotting in hepatocellular carcinoma (HCC) cells treated with TMG, PUG, OE-OGT and siOGT. (B) RALY was measured by qRT-PCR in HCC cells treated with TMG, PUG, OE-OGT and siOGT. (C, D, E). Half-life and quantitative analysis of RALY in HCC cells treated with or without O-GlcNAcylation stimulators (TMG or PUG). HCC cells were transfected with RALYKO-WT (D) or RALYKO-S176A (E) and treated with CHX.

Examining the impact of O-GlcNAcylation on RALY, we treated HCC cells with TMG, PUG and OE-OGT, and found that these stimulators could boost both overall O-GlcNAcylation and RALY protein levels at the same time. Silencing of OGT decreased RALY protein accumulation (Figure 3A). However, the aforementioned stimulus did not influence RALY mRNA expression (Figure 3B). These findings indicate that O-GlcNAcylation regulates RALY protein expression with a transcription-independent mechanism.

Subsequently, it was discovered that TMG could extend the lifespan of the RALY protein (the half-life of RALY in DMSO was approximately 16 hours and 15 hours in Hep3B and Huh7 cells, respectively, but approximately 40 hours and 32 hours in the presence of TMG, respectively). PUG had the same effect (Figure 3C). Furthermore, compared with RALYKO-WT, the S176A mutant also remarkably shortened the RALY half-life, whereas treatment with O-GlcNAcylation stimulators only prolonged the RALYKO-WT half-life period and had a minimal effect on the S176A mutant (Figure 3D and 3E). Collectively, these findings show that O-GlcNAcylation maintains the expression of RALY, possibly through enhanced protein stability.

### O-GlcNAcylation blocks the TRIM27-mediated degradation of RALY

**Figure 4:**
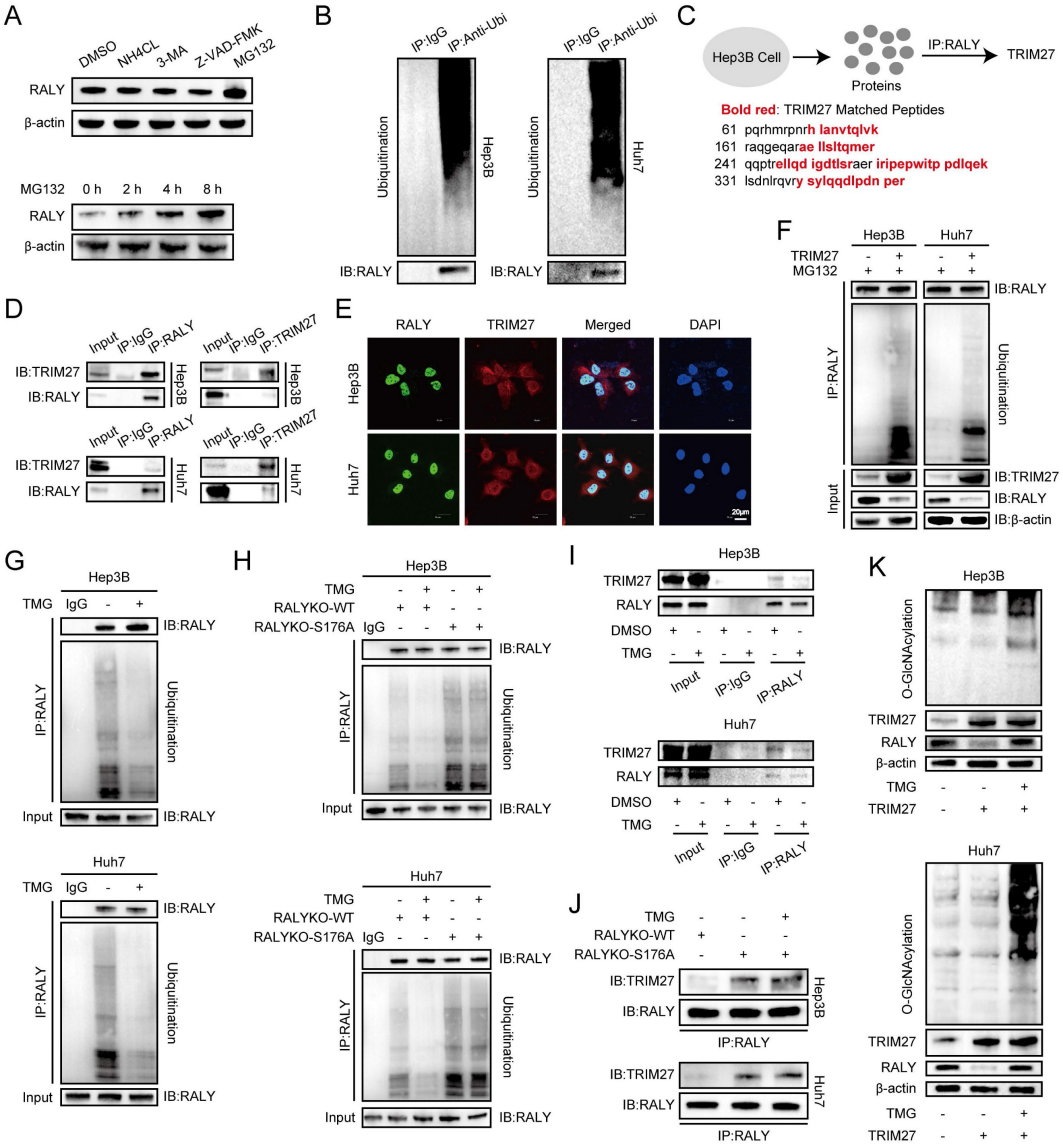
Crosstalk between O-GlcNAcylation and ubiquitination of RALY. (A) The RALY protein level after treated with NH4Cl, 3-MA, Z-VAD-FMK and MG132 in hepatocellular carcinoma (HCC) cells, were analyzed by western blot with indicated antibodies. (B) RALY was examined in total ubiquitinated proteins in HCC cells. (C) Scheme displaying the mass spectrometry procedure used for identifying the specific target TRIM27 of RALY. (D, E) Co-immunoprecipitation (D) and immunostaining assays (E) validation of RALY interaction with TRIM27 *in vitro*. (F) Western blotting indicated the increased level of RALY ubiquitination in HCC cells with TRIM27 overexpression. (G, H) Western blotting indicated the RALY ubiquitination in HCC cells. HCC cells were treated with or without TMG (G); HCC cells were transfected with RALYKO-WT or RALYKO-S176A and treated with TMG (H). (I, J) The level of TRIM27 protein was measured in purified RALY immunoprecipitation samples from HCC cells. HCC cells were treated with or without TMG (I); HCC cells were transfected with RALYKO-WT or RALYKO-S176A and treated with TMG (J). (K) Western blotting showed the RALY protein expression in TRIM27-overexpression HCC cells with TMG treatment.

We next investigated the mechanisms by which O-GlcNAcylation regulates RALY expression. Ubiquitin-proteasome pathway, autophagy-lysosome pathway and caspase-dependent pathway are essential systems responsible for proteolysis in human body 19-21. To clarify which pathway is involved in the proteolysis of RALY, we investigated the effects of the lysosome inhibitors (NH4Cl, 3-MA), the caspase inhibitor Z-VAD-FMK, and the proteasome inhibitor MG132 on the overall protein level of RALY. The findings from our study showed that only MG132 increased the protein accumulation of RALY (Figure 4A), suggesting that RALY is susceptible to degradation by the ubiquitin-proteasome system. The Signal-Seeker Ubiquitination Detection Kit offers a powerful set of tools for purifying ubiquitinated proteins. Our results showed that RALY was present in the total ubiquitinated proteins pulled down by anti-ubiquitination antibodies in HCC cells, suggesting that RALY may be ubiquitinated (Figure 4B). In order to understand the fundamental processes of RALY ubiquitination, we conducted MS after Co-IP with anti-RALY antibodies to search for the E3 ligase from RALY-interacting proteins, which identified the E3 ubiquitin ligase TRIM27 (Figure 4C and Table S2). Co-IP and immunostaining analyses further indicated the interaction of RALY with TRIM27 (Figure 4D and 4E). Prior researches have indicated that TRIM27 mediates the ubiquitination and degradation of other proteins 22, 23. In our analysis, we observed elevated levels of TRIM27 protein in HCC samples when compared to normal tissues (Figure S1). Next, we verified whether TRIM27 can be used to mark RALY for ubiquitination and degradation. Overexpression of TRIM27 resulted in ubiquitination and degradation of RALY in Hep3B and Huh7 cells (Figure 4F). These results suggest that TRIM27 ubiquitinate and degrade RALY.

We found that TRIM27 was responsible for the ubiquitination and degradation of RALY. However, RALY protein maintained high expression levels in HCC cells, avoiding degradation mediated by TRIM27 protein. To further explore the mechanism of RALY overexpression, we then examined whether O-GlcNAcylation affects the RALY ubiquitination. Treatment with TMG significantly decreased the total ubiquitination of RALY (Figure 4G). In addition, the S176A mutant increased RALY ubiquitination more than RALYKO-WT did, while TMG only affected WT ubiquitination and had little impact on S176A (Figure 4H). To study whether high O-GlcNAcylation affects RALY ubiquitination by inhibiting the binding of TRIM27 and RALY, the RALY protein was purified from HCC cells with varying O-GlcNAcylation levels and immunoblotted using an anti-TRIM27 antibody. The expression of RALY-bound TRIM27 was reduced in the TMG-treated group compared to the control group (Figure 4I). Compared to RALYKO-WT, the S176A mutant enhanced the binding of RALY-TRIM27, which was not affected by TMG (Figure 4J). Furthermore, the expression of RALY was examined in HCC cells that had an increased level of TRIM27 and were treated with TMG. The results showed that high levels of O-GlcNAcylation inhibited TRIM27-mediated RALY proteolysis (Figure 4K). In summary, O-GlcNAcylation reverses the ubiquitination-dependent proteolysis of RALY by TRIM27, thus maintaining overexpression of the RALY protein.

### RALY's proliferative effect requires O-GlcNAcylation

**Figure 5:**
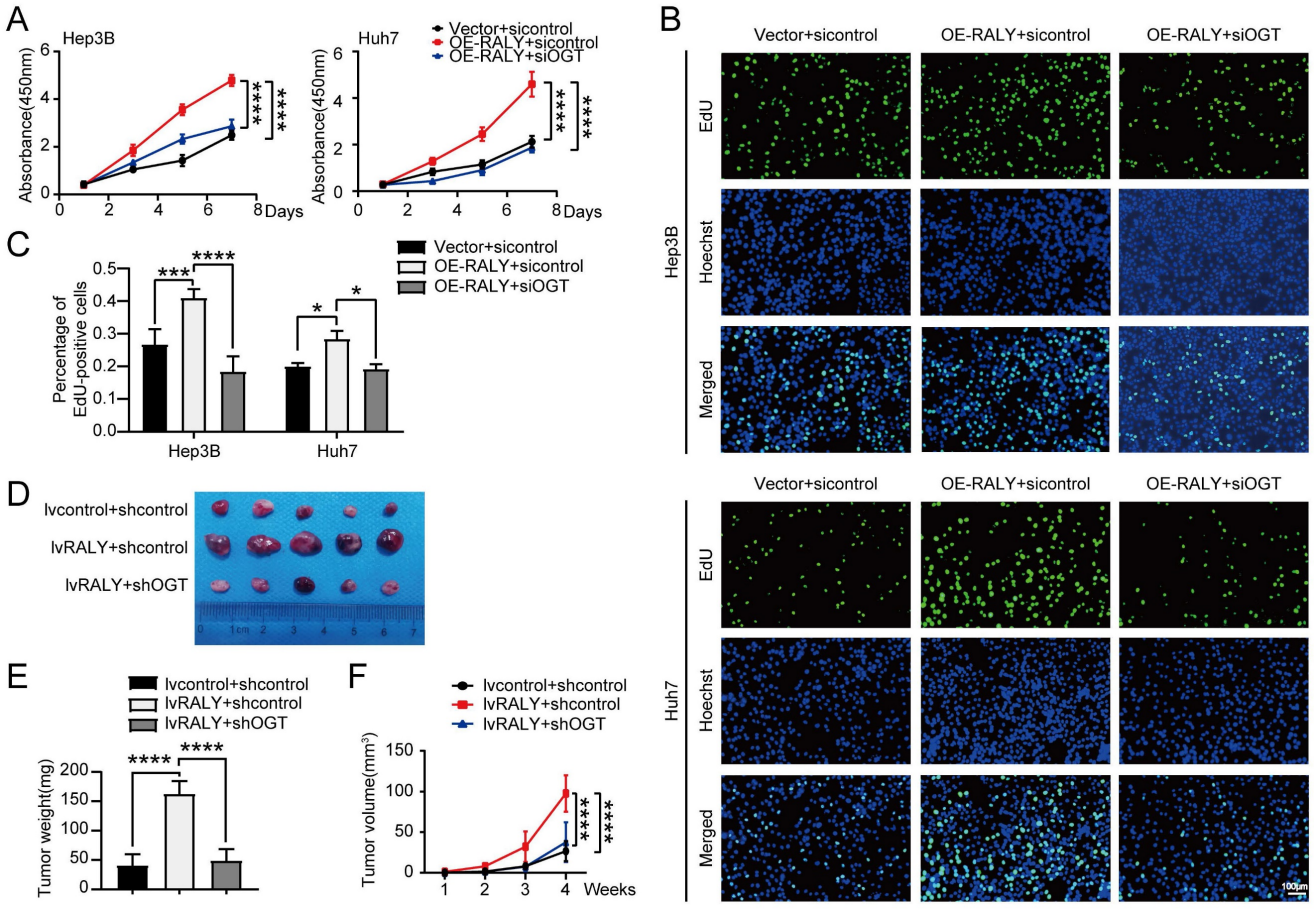
Knockdown of OGT weakened the proliferative effect of RALY overexpression. (A, B) CCK-8 (A) and EdU incorporation assays (B) were performed in hepatocellular carcinoma (HCC) cells for rescue experiments. (C) Statistical analysis of the EdU incorporation assays. (D) The photograph of planted subcutaneous tumors of Huh7 cells infected with the indicated lentivirus. (E) The weight of the tumors was measured at the experimental endpoint. (F) The volumes of tumors were measured every week.

An experiment was carried out to confirm the impact of RALY O-GlcNAcylation on HCC through loss-of-function analysis. CCK-8 and EdU assays demonstrated that OGT silencing reversed the effect of RALY overexpression on cell proliferation (Figure 5A, 5B and 5C). In addition, *in vivo* xenograft experiments showed that depletion of OGT reduced the tumor weight and volume caused by RALY overexpression (Figure 5D, 5E and 5F). Loss-of-function assays reveal that O-GlcNAcylation is essential for the tumor- proliferative effect of RALY.

### RALY interacts with USP22 transcripts and regulates its mRNA export

**Figure 6:**
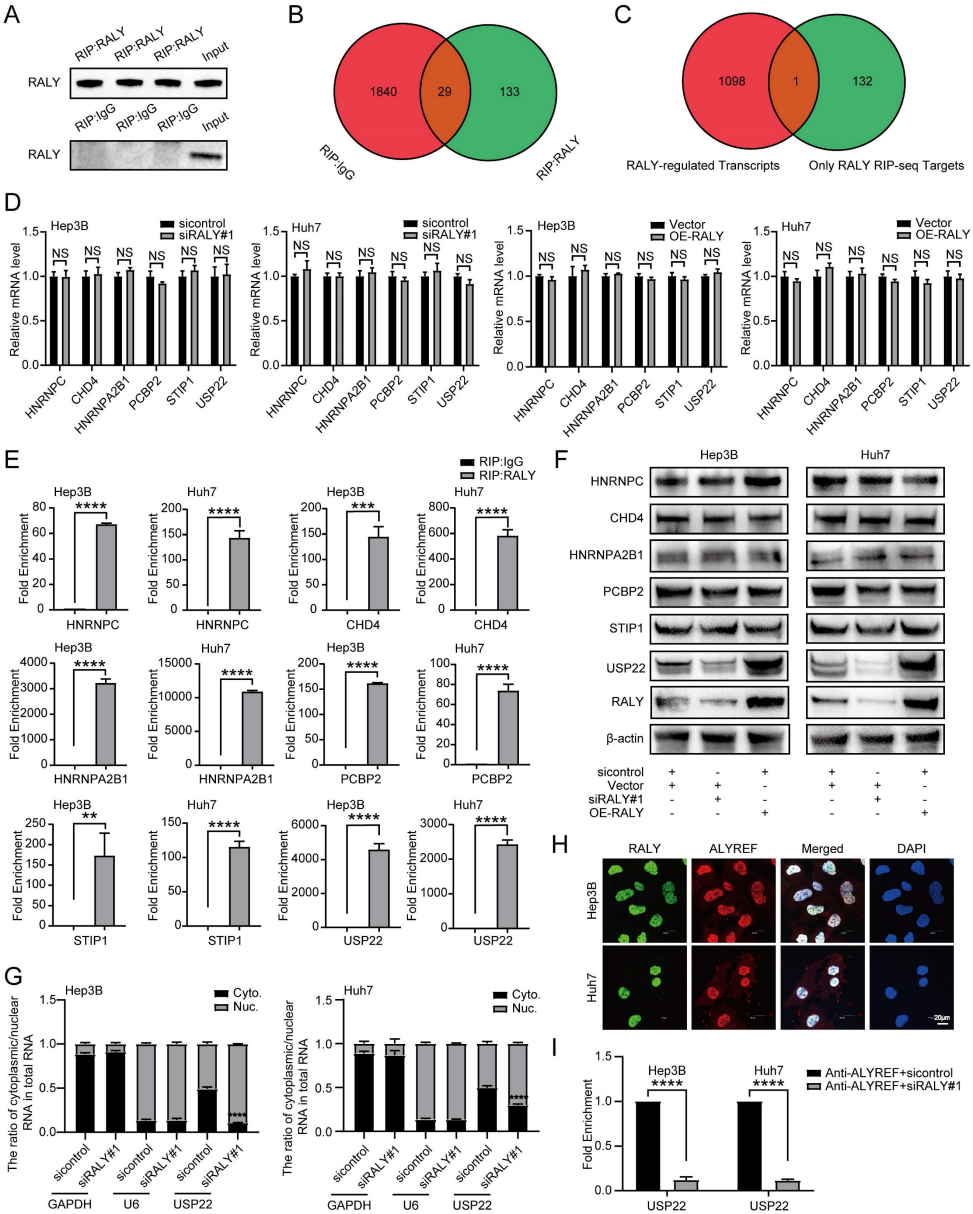
RALY increased the cytoplasmic localization of USP22 mRNAs to enhance their protein expressions. (A) Western blotting showed the efficiency of immunoprecipitation. (B) Venn diagram of transcripts bound by RALY and IgG. (C) Venn diagram of RALY-regulated transcripts and RALY RNA immunoprecipitation (RIP) sequencing targets. (D) The qRT-PCR assays showed the abundance of RALY target RNAs (HNRNPC, CHD4, HNRNPA2B1, PCBP2, STIP1 and USP22) from hepatocellular carcinoma (HCC) cells transfected with siRALY or OE-RALY. (E) RIP-qPCR showed the RNA-binding capacity of RALY in HCC cells. (F) Western blotting showed the protein abundance of RALY target RNAs (HNRNPC, CHD4, HNRNPA2B1, PCBP2, STIP1 and USP22) from HCC cells transfected with siRALY or OE-RALY. (G) Cytoplasmic and nuclear mRNA expression of USP22 in RALY silenced HCC cells. (H) Immunostaining assays validation of RALY interaction with ALYREF *in vitro*. (I) RIP-qPCR verified that the USP22 mRNA-binding capacity of ALYREF was decreased in siRALY HCC cells compared to control.

RBPs interact with RNAs to regulate all aspects of RNA metabolism. To identify RALY-associated transcripts in HCC, we performed RIP using either an anti-RALY antibody or anti-mouse IgG (Figure 6A), followed by RIP-seq. RIP-seq analysis displayed 133 transcripts that were exclusively involved in the immunoprecipitation of anti-RALY and not in the immunoprecipitation of anti-mouse IgG (Figure 6B, Figure S2, and Table S3). To further elucidate the mechanism of RALY-regulated RNA expression, we performed global gene expression profiling with RNA-seq using siRALY-transfected Hep3B cells. In total, A total of 1099 genes showed differential expression (DEGs), with 560 being up-regulated and 539 being down-regulated (Figure S3 and Table S4). Unexpectedly, a comparison between the 133 enriched genes from RIP-seq and 1,099 DEGs from our RNA-seq data yielded very limited overlap of one gene (Figure 6C), suggesting that nucleus-localized RALY had a minor effect on the transcriptional-level accumulation of target RNAs in HCC.

Among these RALY-bound transcripts, we noted several proliferation-related genes, six of which (HNRNPC, CHD4, HNRNPA2B1, PCBP2, STIP1 and USP22 24-29) showed elevated expression in HCC tissues and were linked to unfavorable patient outcomes (Figure S4). Consistent with the RNA-seq findings, the mRNA levels of HNRNPC, CHD4, HNRNPA2B1, PCBP2, STIP1 and USP22 were not significantly influenced by the suppression or enhancement of RALY (Figure 6D). In parallel, RIP-PCR assay results also showed that the above six mRNAs could be specifically bound by RALY in HCC cells (Figure 6E). However, ablation of RALY resulted in a considerable decrease in USP22 protein levels; whereas RALY overexpression increased USP22 protein levels (Figure 6F). These findings suggest that nucleus-localized oncogenic RALY can interact with USP22 mRNA and modulate its protein accumulation rather than transcript levels.

Next, we elucidated the molecular mechanisms underlying the regulation of USP22 by RALY. We hypothesized that RALY might influence USP22 mRNA export. The nuclear/cytoplasmic fractionation experiment revealed a notable decrease in cytoplasmic USP22 mRNA expression in HCC cells transfected with siRALY compared to the control group, implying that RALY positively regulated the cytoplasmic localization of USP22 mRNAs (Figure 6G).

In an effort to gain a deeper comprehension of how RALY controls the cytoplasmic localization of USP22 mRNAs, we searched for RALY-interacting proteins based on the results of MS after Co-IP with anti-RALY antibodies. We found that ALYREF, an adaptor for mRNA export, was present in the RALY-interacting proteins (Figure S5). In addition, the immunostaining assay results demonstrated the nuclear co-localization of ALYREF and RALY of Huh7 and Hep3B cells (Figure 6H). Given that RALY interacts with ALYREF, we hypothesized that RALY enhances the affinity between its target transcripts and ALYREF. To test this hypothesis, we performed RIP of the ALYREF-RNAs complex using an anti-ALYREF antibody in the sicontrol and siRALY groups. Indeed, knockdown of RALY dramatically weakened the association between ALYREF and USP22 mRNAs (Figure 6I). Our data show that RALY enhances the association between ALYREF and USP22 mRNAs, which is a prerequisite for the initiation of nuclear mRNAs export.

### USP22 reversely regulates the siRALY-induced antitumor-promoting effects of HCC

**Figure 7:**
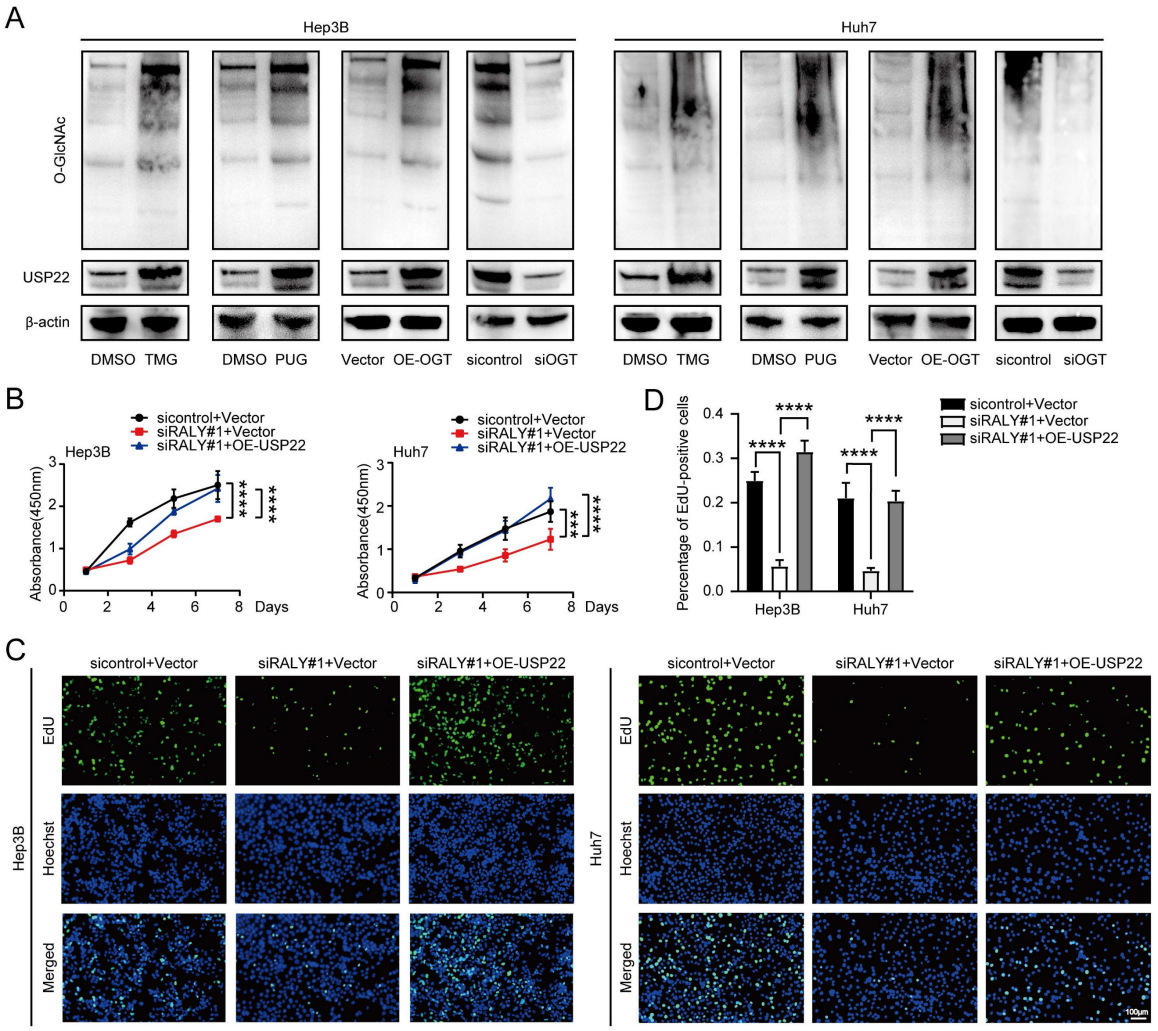
RALY exerted its biological functions by promoting USP22 expression in hepatocellular carcinoma (HCC) cells. (A) Western blotting showed the expression of USP22 in two HCC cell lines by stimulation and block of O-GlcNAcylation. (B, C) Cell proliferative capacity was determined after cell transfection by CCK-8 (B) and EdU incorporation assays (C). (D) Statistical analysis of the EdU incorporation assays.

USP22, a downstream component of RALY, was found to play a crucial part in the proliferation of HCC in my research (Figure S6). Western blotting revealed that USP22 expression was also regulated by global O-GlcNAcylation (Figure 7A). Next, we determined whether reconstituting the expression of USP22 in HCC cells depleted of RALY could reverse the reduced proliferative ability of HCC cells *in vitro*. We observed that overexpression of USP22 remarkably strengthened the proliferative capacity of RALY-depleted HCC cells, as revealed by CCK-8 (Figure 7B) and EdU analyses (Figure 7C and 7D). Together, these results reveal that RALY promotes HCC cells proliferation through USP22.

### RALY-PROTACs induce RALY protein degradation and decrease the proliferation of HCC cells

In recent decades, bi-functional degrader molecules, also known as proteolysis-targeting chimeras (PROTACs), have become a prominent approach for degrading disease-causing proteins, bringing targeted cancer therapy into a new era. This technology recruits a targeted protein in proximity to an E3 ubiquitin ligase to trigger protein ubiquitination and proteolysis via the ubiquitin-proteasome system 30. However, PROTACs applications are limited in part because current small-molecule PROTAC technologies cannot target proteins that do not contain small-molecule binders. Compared with traditional PROTACs, p-PROTACs is simpler in design and synthesis and can target undruggable proteins because of the presence of peptides that specifically bind to targeted proteins 31.

**Figure 8:**
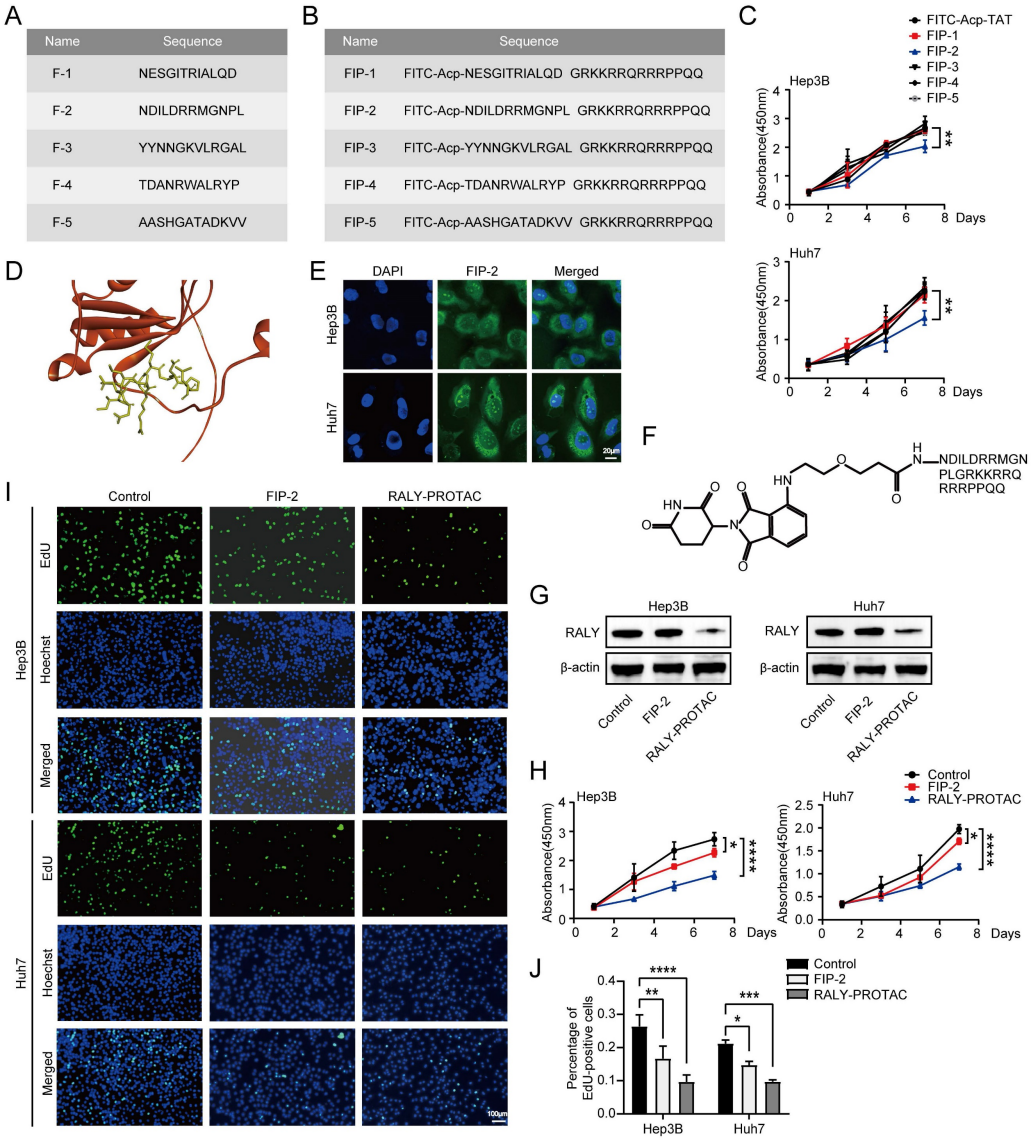
Peptide‑based proteolysis-targeting chimeras (p-PROTAC) degrader of RALY. (A) Name and sequence of peptides targeting RALY protein. (B) Name and sequence of peptides conjugated with FITC and TAT sequence at the N-terminus and C-terminus, respectively. (C) CCK-8 showed the growth rate of hepatocellular carcinoma (HCC) cells treated with five screened peptides. (D) The optimized binding modes with lowest binding energy generated by ClusPro web server and interaction between RALY and F-2, Red: protein; Yellow: F-2. (E) Cellular localization of FITC labeled FIP-2 (green) by immunostaining assays. (F) Structure of RALY-PROTAC. (G) Western blotting showed the expression of RALY protein in HCC cells treated with FIP-2 and RALY-PROTAC. (H, I) CCK-8 (H) and EdU incorporation assays (I) of HCC cells treated with FIP-2 and RALY-PROTAC. (J) Statistical analysis of the EdU incorporation assays.

To develop p-PROTACs of RALY, we screened and identified peptides using phage display libraries based on phage screening. Five RALY-targeting peptides were identified (Figure 8A). For the peptides to successfully penetrate the cell membrane and achieve high intracellular concentrations, we chose the cell membrane-penetrating peptide TAT 32, 33 as a transporter to help our small-molecule compounds enter the cell. Therefore, we coupled the TAT sequence and FITC with the peptides and obtained five synthesized peptides with membrane penetration properties (Figure 8B). We confirmed the inhibitory effects of the five synthesized peptides on HCC cells. FIP-2 suppressed HCC cell growth (Figure 8C). The molecular docking results showed that F-2 had the affinity for RALY (Figure 8D) 34, 35. Moreover, fluorescence confocal microscopy showed that FITC-labeled FIP-2 was able to penetrate the nucleus effectively (Figure 8E). In summary, these data indicated that FIP-2 was selected as the peptide for the development of p-PROTACs. A novel peptide-based PROTAC was formed by coupling FIP-2 with Pomalidomide-PEG1-C2-COOH and was named RALY-PROTAC (Figure 8F). Western blotting results revealed that RALY-PROTAC significantly degraded the RALY protein (Figure 8G). As shown in Figure 8H-8J, HCC cells proliferation decreased after treatment with RALY-PROTACs. Collectively, RALY-PROTAC targeting RALY degradation may be a hopeful approach for suppressing HCC.

## Discussion

Increasing evidence suggests that RBPs have multiple important biological functions 36-38. However, their role in HCC remains largely unexplored. RALY, an RBP, exhibits tumor-promoting effects in HCC. A prior investigation examined the impact of RALY on controlling the MTA1 splicing switch 39; however, the expression regulatory mechanisms and functions of RALY in HCC are still not well understood. This research shows that RALY, which is site-specifically O-GlcNAcylated, is notably resistant to proteolysis by ubiquitination. Stable RALY promotes tumor growth by regulating the ALYREF-dependent nuclear export of USP22 mRNA. These findings not only show the upstream mechanism of overexpression of RALY, but also reveal the specific downstream pathways through which RALY regulates HCC cell proliferation.

O-GlcNAcylation alters the activity, stability, and interaction of functional target proteins in HCC 40. O-GlcNAc modifying Ser263 of YTHDF2 inhibits YTHDF2 proteolysis by inhibiting its degradation and promoting hepatocellular carcinogenesis 41. OGT-mediated O-GlcNAcylation promotes the nuclear localization of SPOP in HCC cells, leading to the alleviation of Nogo-B protein ubiquitination 42. O-GlcNAc-modified RACK1 enhances ribosome binding and interaction with PRKCB, thereby increasing the translation of potent oncogenes in hepatoma cells 43. Here, we show that O-GlcNAcylation maintains the protein stability of RALY, and a mutation at Ser176 does not completely eliminate the O-GlcNAcylation level, indicating that other O-GlcNAc modification sites have not been found. Further efforts are required to obtain a whole map of O-GlcNAcylated RALY sites.

It is increasingly accepted that RBPs are essential mediators of gene expression that potently and ubiquitously mediate all stages of their RNA partner's lifespans, including splicing, transport, translation, and stability 37, 44, 45. Recent studies have demonstrated that RALY, a multifunctional RBP, interacts with several mRNAs to regulate the expression of these transcripts.

RALY binds to the mRNA of the proliferation marker E2F1 and regulates its stability, thereby affecting the proliferative ability of cells 46. Moreover, RALY modulates alternative splicing of mRNA, which leads to RNA complexity and protein diversity in cancer 39. In addition to mRNA, RALY recruits pri-miRNAs to the microprocessor complex, thereby promoting their processing 47. As a long non-coding RNA, the stability of TERRA is reportedly regulated by RALY 48. A previous study filled this gap with a comprehensive global analysis of RALY-related RNAs 49; however, the role of RALY in regulating RNA post-transcriptional control in HCC remains elusive. Thus, the RALY-binding partners identified in this research may offer new mechanistic insights into the regulation of gene expression during HCC progression. We identify USP22 mRNA as a downstream target of RALY, which plays a regulatory role in the nuclear export of USP22 mRNA rather than in RNA processing. Conserved transcription and export is a multi-subunit protein complex that participates in mRNA export 50, 51. ALYREF is the only protein that harbors a canonical RNA-binding motif in the transcription-export complex and is known to directly interact with RNAs 52-54. Furthermore, ALYREF has the ability to attach to CDKN1A mRNA and aid in the movement of CDKN1A mRNA from the nucleus to the cytoplasm 55. Here, we show that RALY interacts directly with ALYREF and promotes USP22 mRNA nuclear export by transferring it to ALYREF.

Due to the fact that RALY serves as a key oncogene, the biologically targeted degradation of RALY could become an effective therapeutic strategy. PROTAC, a novel therapeutic modality, targets targeted proteins to the E3 ubiquitin ligase in order to break them down. Unlike PROTAC, p-PROTAC has the benefits of increased specificity and reduced toxicity 56. Wang et al. conjugated pomalidomide with a FOXM1-binding antagonistic peptide to synthesize a novel FOXM1-targeted p-PROTAC that successfully suppressed the viability, migration, and invasion of various cancers 57. Here, we synthetize novel RALY-targeting p-PROTACs that can effectively degrade RALY proteins and inhibit the malignant phenotype of HCC cells.

In conclusion, we reveal the effect of O-GlcNAcylation on the RALY protein stability and the molecular mechanism of RALY on the posttranscriptional regulation of USP22 in HCC. Specifically, O-GlcNAcylation inhibits RALY proteolysis and RALY is required for the ALYREF-dependent export of USP22 mRNA. Thus, targeting RALY degradation to inhibit cancer progression may be a new therapeutic strategy for HCC.

## Supplementary Material

Supplementary figures and tables.
