# Supplementary material for: O-GlcNAcylated RALY Contributes to Hepatocellular Carcinoma Cells Proliferation by Regulating USP22 mRNA Nuclear Export
Source: Int J Biol Sci. 2024 Jul 1;20(9):3675–90. doi: 10.7150/ijbs.97397 (PMC11234212; doi:10.7150/ijbs.97397)
Supplement: Supplementary file 1 — Supplementary figures and tables. [file ijbsv20p3675s1.zip › Supplementary figure.pdf]

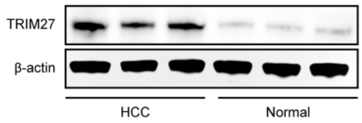

Figure S1

Western blotting of the protein levels of TRIM27 in hepatocellular carcinoma and normal adjacent tissues.

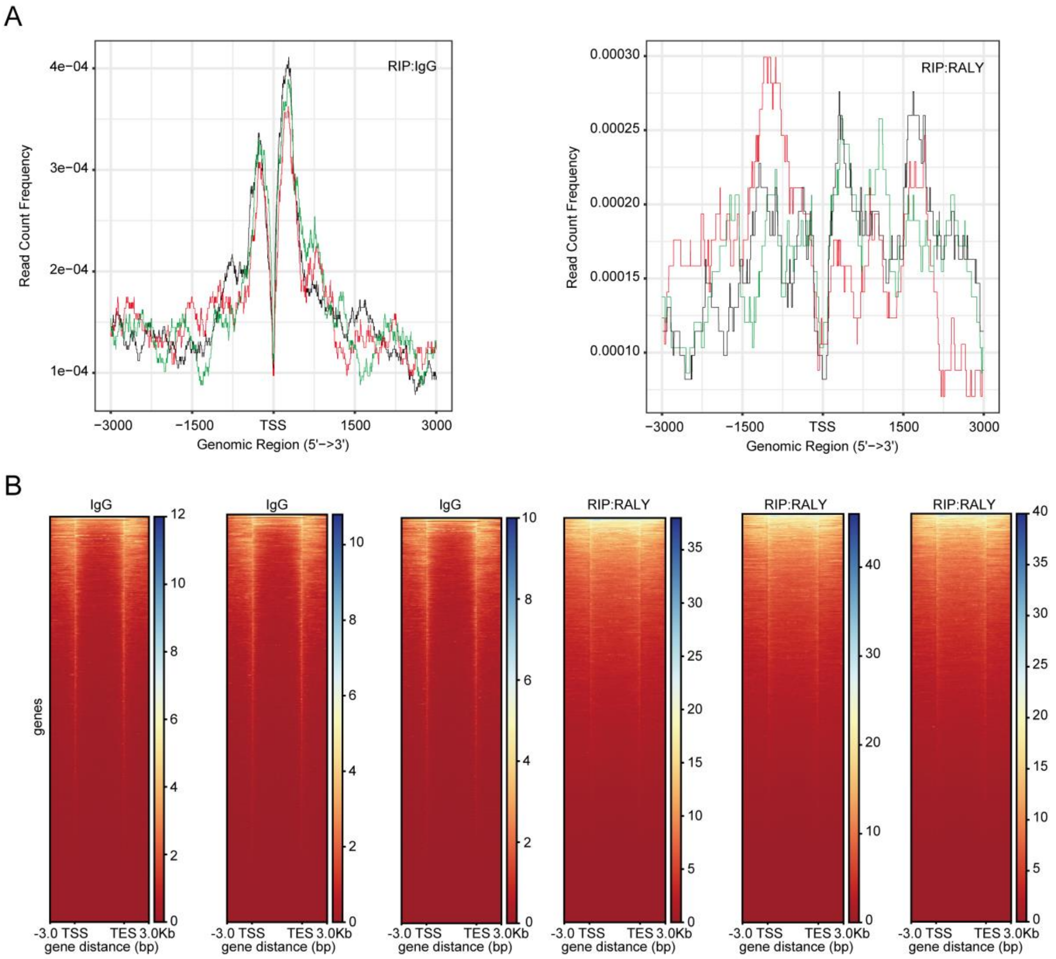

Figure S2

(A) RALY-binding transcripts identified through RIP sequencing (RIP-seq). Negative control: IgG. (B) Heatmap analysis of RIP-seq data for RALY and IgG control.

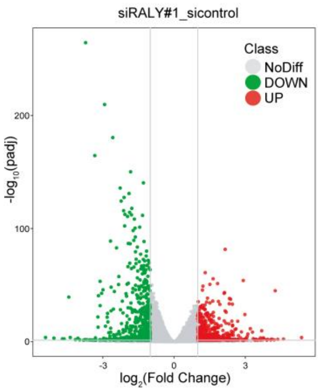

Figure S3

The volcano plot of RALY-silencing transcriptome profiling. Differentially expressed genes (DEGs) were identified based on p value < 0.05 and $|\log_2(\text{Fold Change})| > 1$. Genes significantly up-regulated (red) or down-regulated (green) upon RALY silencing were highlighted.

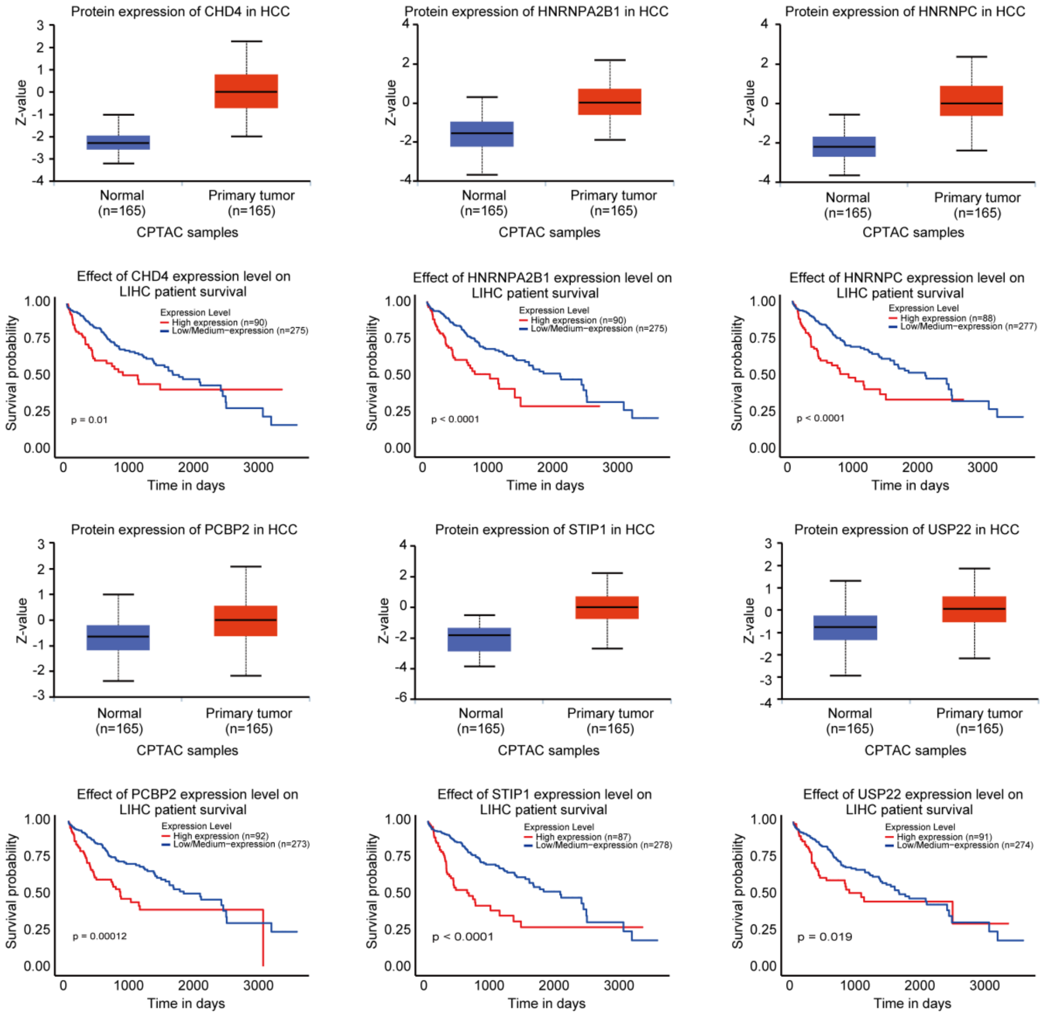

Figure S4

HNRNPC, CHD4, HNRNPA2B1, PCBP2, STIP1 and USP22 protein levels in hepatocellular carcinoma (HCC) and normal adjacent tissues predicted by an online database (UALCAN); Kaplan-Meier analysis was used to detect the survival rate of HCC patients in an online database (UALCAN).

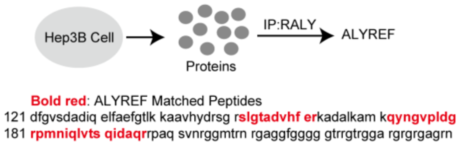

Figure S5

The Scheme displaying the mass spectrometry procedure used for identifying the specific target ALYREF of RALY.

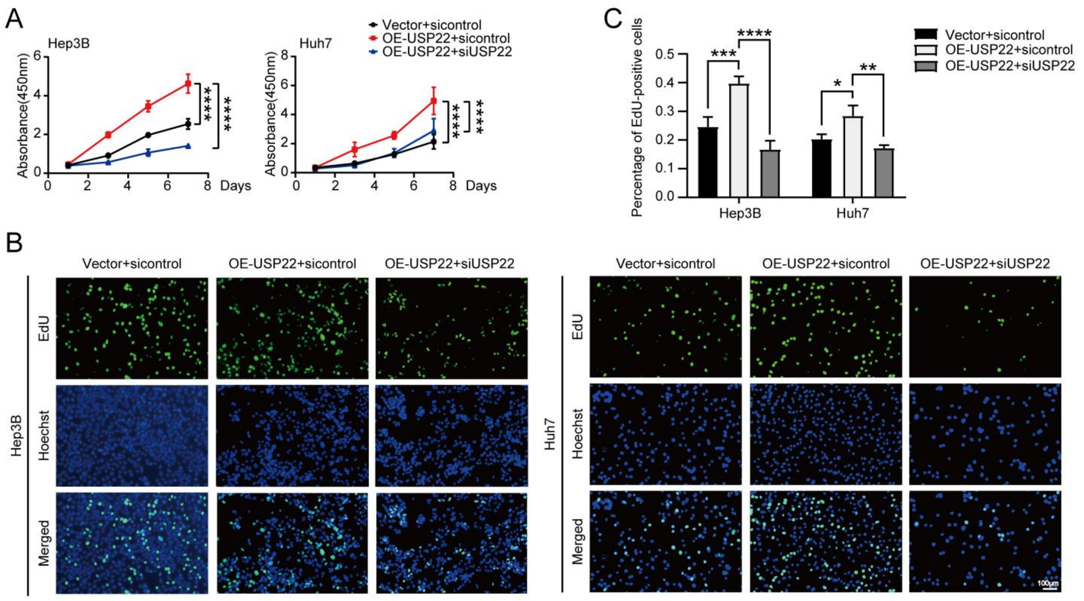

Figure S6

USP22 promoted hepatocellular carcinoma (HCC) cells proliferation in vitro. (A) CCK-8 was utilized to assess cell proliferation capacity of HCC cells. (B) Cell proliferation capacity of HCC cells was tested by EdU incorporation assays. (C) Statistical analysis of the EdU incorporation assays.
